# The cardioprotective effects of the new crystal form of puerarin in isoproterenol-induced myocardial ischemia rats based on metabolomics

**DOI:** 10.1038/s41598-020-74246-y

**Published:** 2020-10-20

**Authors:** Yuzhi Zhou, Mengru Li, Jia Song, Yongqiang Shi, Xuemei Qin, Zhaolin Gao, Yang Lv, Guanhua Du

**Affiliations:** 1 Key Laboratory of Drug Target Research and Drug Screen, Institute of Materia Medica, Chinese Academy of Medical Sciences and Peking Union Medical College, 2A Nan Wei Road, Beijing, 100050 China; 2 Shandong Province Key Laboratory of Polymorph Drugs, Shandong Yikang Pharmaceutical Co. Ltd, No. 3288, Yikang Avenue, Tengzhou, 277513 China; 3 Modern Research Center for Traditional Chinese Medicine, Shanxi University, No. 92, Wucheng Road, Taiyuan, 030006 China

**Keywords:** Metabolomics, Biomarkers, Pharmacology

## Abstract

Puerarin has shown unique pharmacological effects on myocardial ischemia (MI). Changing the crystal form is an effective approach to improve the cardioprotective effects of puerarin. However, the mechanisms of the new crystal form of puerarin are unclear. In this study, an electrocardiogram, echocardiography, cardiac marker enzymatic activity, oxidative stress indices, and myocardial histology analysis of cardiac tissues were performed to evaluate the cardioprotective effects of the new crystal form of puerarin. Moreover, serum and cardiac tissue metabolomics based on nuclear magnetic resonance (NMR) were used to investigate the potential mechanism of the new crystal form. The results indicated that the new crystal form of puerarin (30 mg/kg) could improve oxidative stress indices, and these improvements were similar to those of the original crystal form of puerarin (120 mg/kg). The new crystal form of puerarin (30 mg/kg) could effectively improve the activities of cardiac marker enzymes, and the improvement effects were better than those of the original crystal form (120 mg/kg). Moreover, metabolomics analysis showed that amino acid metabolism, oxidative stress and energy metabolism were disturbed after MI and could be improved by puerarin. These results demonstrated that the new crystal form of puerarin was effective in treating MI.

## Introduction

Myocardial ischemia (MI), one of the most serious threats to human health, is a pathological state of the heart caused by insufficient blood supply, that leads to a reduced oxygen supply in the heart tissues and abnormal myocardial metabolism^1^. It is currently still a significant research subject in the field of medicine due to both its high prevalence and mortality^2^. A large number of patients suffer from other diseases associated with myocardial ischemia, such as angina, heart failure and coronary artery atherosclerosis, leading to a very large economic burden and gradual decline in quality of life^3,4^. Therefore, in addition to lifestyle modification, there is a need to develop novel treatment methods to reduce the burden of MI in society^4^.

In order to treat MI, natural extracts isolated from traditional Chinese medicine (TCM) have received increasing attention. Puerarin, a major bioactive ingredient isolated from the roots of** Pueraria lobate (Willd.) Ohwi** (“Gegen” in TCM), has shown unique pharmacological effects on ischemic heart disease^5,6^. Myocardial protection involves various factors, such as vasodilation, oxidative stress, heart rate, and cardiomyocyte enzymes^5^. However, the low oral bioavailability and poor water solubility of puerarin has limited its administration and has led to poor efficacy. These disadvantages indicate that frequent administration of a high dose of puerarin is needed, which leads to adverse effects^6,7^. At present, the study of pharmaceutical crystallography has attracted widespread attention to solve the problems of poor solubility and low oral bioavailability of drugs^8^. Thus, changing the crystal form of puerarin is an effective way to improve its treatment efficacy. A previous study showed that puerarin-V had cardioprotective effects on isoproterenol-induced myocardial infarction in mice, and puerarin-V had more advantages than the other modified crystal forms of puerarin, including better absorption and higher plasma drug concentration, solubility and stability^9^.

In numerous experimental studies, isoproterenol (ISO) was used to induce MI in rats^10–13^, which could cause pathological changes in the rat heart that were similar to MI^4,12^. Electrocardiogram and echocardiography, which directly reflect several essential capacities of cardiac function, have been widely used in the diagnosis of MI and the assessment of drugs against this disease^14^. Additionally, various studies have demonstrated that endogenous antioxidants and cardiac enzymes were significantly changed in an animal model of MI^15–17^, and representative cardiac enzymes have very high specificity and sensitivity in clinical diagnosis^17^. Thus, the combination of electrocardiogram, echocardiography and serum biochemical assays have been used to investigate the cardioprotective effects of the new crystal form of puerarin.

Metabolomics is a novel biological approach and a breakthrough technology for the systematic analysis of metabolites to understand metabolic alterations^18^. With a relatively short history, it has been widely applied in various research fields, such as to study drug toxicity^19,20^, disease diagnosis^21,22^, and natural product discovery^23^. Nuclear magnetic resonance (NMR) detection not only can be completed in a short time, which is critical for maintaining the original properties of a sample during the test period, but also provides a very large amount of information on the arrangement of metabolites from only one measurement^1^. The application of NMR-based metabolomics might be an ideal approach to identify endogenous metabolites, which can further explain the overall changes from the perspective of metabolic networks and evaluate the changes in biomarker metabolites. In this study, NMR analysis of both the serum and heart tissues was used to investigate the protective effects of the new crystal form of puerarin and its potential mechanism.

In this study, we investigated the cardioprotective effects of the new crystal form of puerarin (puerarin-V), and clarified its potential mechanisms based on heart and serum metabolomics in isoproterenol-induced myocardial ischemia rats.

## Materials and methods

### Chemicals and reagents

The new crystal form (puerarin-V, purity > 99%) and the original crystal form of puerarin (purity > 97%) were provided by prof. LV Yang from *the Institute of Materia Medica in Chinese Academy of medical sciences (Beijing, China)*, then kept in the laboratory at *Modern Research Center for Traditional Chinese Medicine, Shanxi University,* Taiyuan. China. ISO was purchased from Santa Cruz Biotechnology, USA. Propranolol hydrochloride (> 99% purity) was acquired from Tianjin Li sheng Pharmaceutical Co., Ltd (Tianjin, China). Cardiac troponin I (cTnI), cardiac troponin T (cTnT) kits were obtained from Sangon Biotech Co., Ltd. (Shanghai, China). Creatine kinase (CK), Lactate dehydrogenase (LDH), Superoxide dismutase (SOD), Malondialdehyde (MDA) assay kits were acquired from Jiancheng Bioengineering Research Institute (Nanjing, China). Sodium chloride injection was purchased from pharmaceutical of Hai Peng Co., Ltd. (Shijiazhuang, China). D_2_O (99.9%) and CD_3_OD were purchased from Norell (Landisville, NJ, USA). 3-(Tri-methy-silyl) propionate-2,2,3,3-d4 acid sodium salt (TSP) was bought from Cambridge Isotope. Ultrapure water was prepared using a Millipore water-purification system (Millipore, MA, USA).

### Experimental animals

A total of 60 male Sprague–Dawley (SD) rats, weighing 180–200 g, were acquired from Beijing Vital River Laboratories (Beijing, China, No. SCXK2018-0011). And all rats were kept at standard laboratory conditions (25 ± 1 °C temperatures and 55 ± 5% relative humidity) with a 12 h light/dark cycle, and food and water ad libitum. All animals handling and treatments were conducted in accordance with the National Guidelines for Experimental Animals Welfare (China, 2006), and experiments had also obtained approval from the Animal Ethics Committee of Shanxi University (SXULL2018025). After 1 week of acclimation, all SD rats were randomly divided into six groups (*n* = 10 per group): the control group (Control), the ISO-damaged group (ISO) and the different drug administration groups including the propranolol group (P), the original crystal form of puerarin group (Pue-Y), the low and high dose of the new crystal form of puerarin groups (Pue-L and Pue-H). In this study, the rats in model group and the drug-administrated groups were administrated ISO (85 mg/kg) by intraperitoneal injection (i.p) on final two consecutive days to establish the model of MI^24^, while control rats were injected the equivalent volume of saline. And for control and model groups, the rats were intragastric (i.g) administrated starch by the new type of gavage needle (CN201020240833.2), and other rats were respectively administered (i.g) the new crystal form of puerarin (30, 120 mg/kg/day), the original crystal form (120 mg/kg/day) and the propranolol(15 mg/kg/day) for 7 days.

### Electrocardiogram detection

On the final day of the experiment, all rats were anesthetized with 3% chloral hydrate (i.p, 0.6 ml/100 g) and then monitored for heart rate and electrocardiogram using the Biopac MP100 data acquisition system (Biopac Systems, Inc., Santa Barbara, CA) under general anesthesia. Electrocardiogram signal activity was recorded for at least 25 min with a sampling frequency of 500 Hz under anesthesia, using disposable electrodes attached to the thorax of the rat. Therefore, the mean and standard deviation (SD) of the waves for each individual electrocardiogram signal was calculated.

### Echocardiography detection

After the electrocardiogram, the echocardiography of all rats was performed with a GE Vivid 7 (GE Health Medical, Milwaukee, WI, USA). Left ventricular (LV) ejection fraction (EF%) and fractional shortening (FS%) were taken as measurements of LV systolic function. Measurements were obtained from two-dimensional and M-mode images of the left ventricle from the parasternal short-axis view^25,26^. All of the echocardiography was performed by the same investigator, who was blinded to the treatments.

### Sample collection

After the end of the echocardiography test, all rats were anesthetized under 3% chloral hydrate (i.p, 0.6 ml/100 g). The blood samples of all rats were collected by abdominal aorta and allowed to clot for 30–40 min at room temperature (25 ± 1 °C). Serum was subsequently isolated by centrifugation at 3500 rpm for 15 min at 4 °C and stored at − 80 °C for biochemical assays. After blood collection, all SD rats were sacrificed by decapitation. Heart tissues were quickly removed and weighed the weight, and lower portions of the myocardial tissues were cut and fixed in 10% buffered formalin solution, then other samples frozen immediately in liquid nitrogen and stored at − 80 °C for further analysis.

### Heart weight index determination

The final body weight of all rats was measured before electrocardiogram test. After rats were euthanized and heart tissues were carefully separated, then weighed with the electronic balance. The heart weight index (HWI) was calculated by the following equation: HWI (mg/g) = heart weight (mg)/body weight (g).

### Biochemical assays in serum

Biochemical indicators of myocardial damage and oxidative stress were used to diagnose the MI, including CK, LDH, cTnT, cTnI, SOD and MDA. These indices in serum were measured using commercial reagent kits according to the manufacturer's protocols, respectively.

### Histopathological examination

Histopathological assessments were performed by using formalin-fixed heart tissues. And myocardial tissues were sectioned at 4–5 μm, then stained with hematoxylin–eosin (H&E). Fixed slides were examined and images were acquired by a light microscope (Olympus, Japan).

### Sample preparation for NMR analysis

For the NMR analysis of serum, 450µL of the thawed serum sample was mixed with 350µL D_2_O (0.2 mol/L Na_2_HPO_4_ and0.2 mol/L NaH_2_PO_4_, PH7.40, containing 0.1%TSP), and centrifuged at 13,000 rpm for 20 min at 4 °C, then 600μL supernatant of the sample was transferred to 5 mm NMR tubes for NMR analysis. For NMR analysis of heart tissues, 200 mg of tissue was homogenized in a low temperature about 3 min, followed by adding 300 μL methanol and 600 μL ultra-pure water. Then the mixture was centrifuged at 3500 rpm for 15 min. Next, the supernatants were blew to near dry by N_2_, and the residue was re-dissolved in 750 μL of phosphate buffer (0.2 mol/L Na_2_HPO_4_ and 0.2 mol/L NaH_2_PO_4_, in 10% D_2_O, PH 7.40, containing 1% TSP, as an internal NMR chemical shift standard), then the mixture was centrifuged at 13,000 rpm for 20 min at 4 °C after vortexed. Subsequently, aliquots of the supernatant (600 μL) were transferred into a 5 mm NMR tube for NMR analysis.

### NMR spectroscopy analysis

All ^1^H NMR spectra were recorded on a Bruker 600.13 MHz AVANCE III NMR spectrometer (Bruker BioSpin, Germany).The serum spectra were analyzed by using a pulse sequence of standard Carr-Purcell-Meibom-Gill (CPMG), and measured with 64 scans requiring a 2.7263 s acquisition time with the following parameters: spectral width of 12,019.2 Hz, spectral size of 65,536 points, and a relaxation time of 1.0 s. The spectra of heart samples were analyzed by the noesygppr1d pulse, and the following parameters were collected: a spectral width of 12,345.7 Hz, a relaxation delay of 1.0 s, spectral size of 65,536 points, and number of scans was 64.

### ^1^H NMR data processing and multivariate data analysis

The acquired ^1^H NMR spectra of all samples were manually phased, corrected baseline by using the MestReNova 8.0.1 software (Mestrelab Research S.L, Spain). And the chemical shifts of spectra from serum were referenced to the creatinine (δ 3.04), while that in heart tissues were referenced to the TSP (δ 0.00). The spectral regions of δ 0.70–9.00 ppm subsequently were integrated at equal width of 0.01 ppm, and the regions contained water signals δ 4.50–5.10 in serum and δ 4.60–5.10 in heart samples were removed to eliminate variations. The remaining spectra were regarded as variables and then normalized to the total sum of integrals for subsequent multivariate statistical analysis. Detailed ^1^H NMR spectra were listed in Supplementary File.

Multivariate statistical analysis was conducted to analyze the NMR data from all samples by the software SIMCA-P 14.0 software (Umetrics, Sweden). Unsupervised principal component analysis (PCA) was used to give an overview of the metabolic patterns and trends, and to detect outliners among samples^1,27^. Partial least squares-discriminant analysis (PLS-DA) and orthogonal projection to latent structure discriminate analysis (OPLS-DA) were subsequently performed to analyze and identify the discrimination of the six experimental groups^14,27^. The total explained variables R^2^X and the model predictability Q^2^ were commonly used to describe the quality of the model. And the validation of these models was further performed by rigorous permutation tests with 200 times. In the OPLS-DA model, the results between two groups were visualized in the forms of score plot and S-plot to show the group clusters contributing to the classification. The important metabolites were filtered by combing with variable importance (VIP, VIP > 1) and t test (*p* < 0.05) as potential biomarkers related to the MI.

### Statistical analysis

In this study, statistical analysis was conducted with Graph Pad Prism 7.0 software (San Diego, CA, USA). All data of various groups were presented as mean ± S.D, and the intergroup comparisons were evaluated by performing with turkey-test and One-way ANOVA. And the results were considered as statistically significant when *t*-tests and *p* values less than 0.05.

## Results

### Effects of puerarin on electrocardiogram

**Figure 1:**
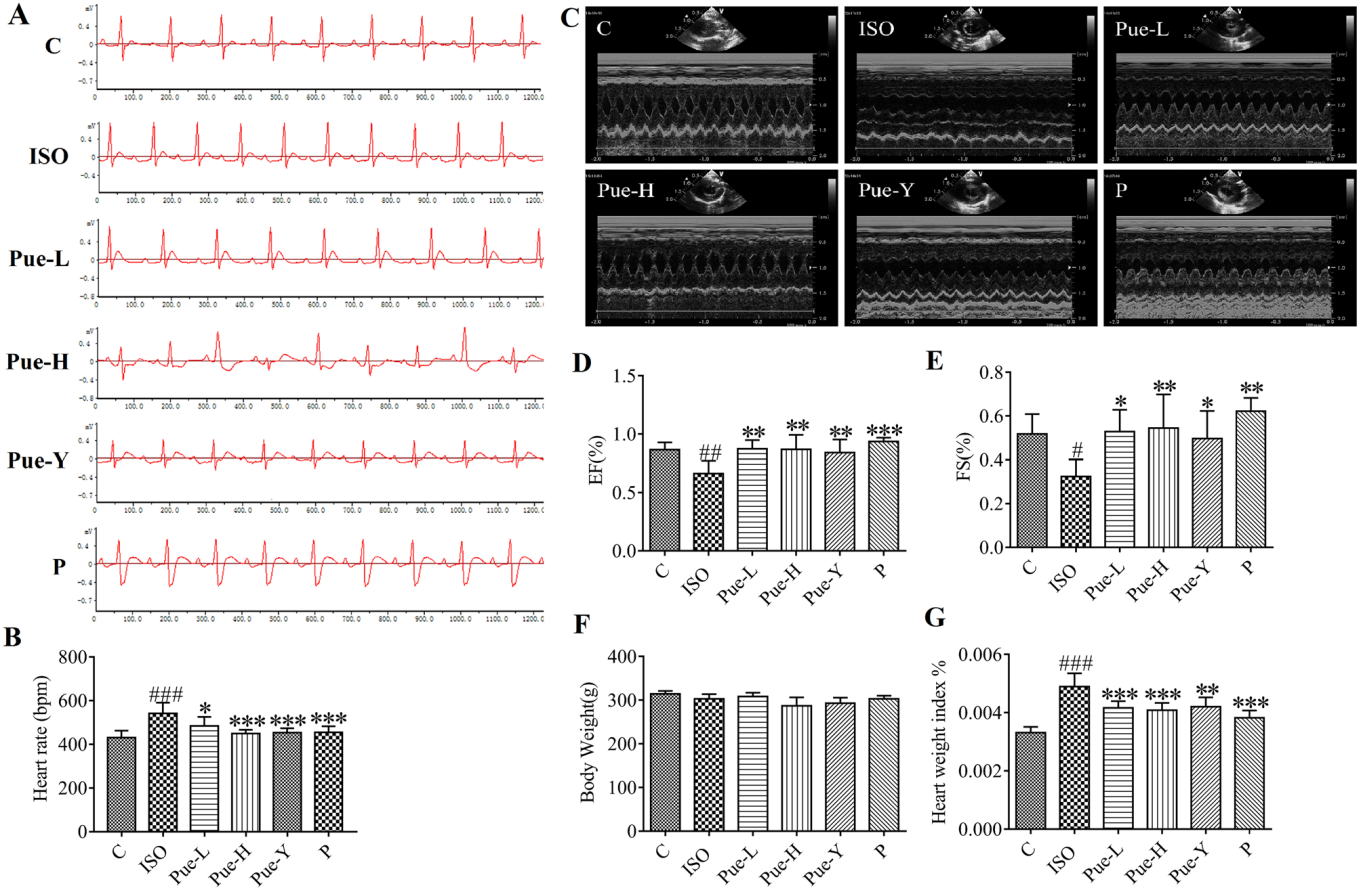
Effects of the puerarin pretreatment on heart. (**A**) Electrocardiogram of six groups. (**B**) The effects on heart rate. (**C**) Two-dimensional and M-mode short-axis images. (**D**,**E**) Echocardiography parameters (EF% and FS%). (**F**,**G**) Final body weight and heart weight index (HWI). HWI (%) = Heart weight /Body weight *100%. Data was analyzed by using one-way ANOVA. Data are shown as Mean ± SD, (n = 6). #*p* < 0.05, ##*p* < 0.01, ###*p* < 0.001 vs control group, *p < 0.05, **p < 0.01, ***p < 0.001 vs ISO group.

In order to evaluate the efficacy of puerarin on ISO-induced alterations to the electrical activity of the heart, electrocardiograms were recorded (Fig. 1A). As depicted in Fig. 1B, the heart rates of model rats were significantly elevated compared with those in the control group (*p* < 0.001). Compared with the rats in the model group, the heart rates were significantly decreased in the groups pre-administered puerarin-V (120 mg/kg, *p* < 0.001), the original crystals of puerarin (120 mg/kg, *p* < 0.001) and propranolol (15 mg/kg, *p* < 0.001) and puerarin-V (30 mg/kg, *p* < 0.05).

### Effect of puerarin on echocardiography

Figure 1C showed a two-dimensional image of the left ventricle from the model group, which is clearly different from the left ventricle from the other groups. Figure 1D,E show that the EF% (*p* < 0.01) and FS% (*p* < 0.05) were notably reduced in the ISO group compared with the control group, which indicated that the myocardial ischemia model was replicated successfully. Compared with the model group, the Pue-L group (30 mg/kg) showed similar improved effects as the Pue-Y group (120 mg/kg), which increased both the EF% (*p* < 0.01) and FS% (*p* < 0.05) indices. Moreover, the new crystal form (120 mg/kg) showed better treatment effects than the original crystal form (120 mg/kg) in the alteration of FS%. These data suggested that administration of the new crystal form of puerarin could protect cardiac function in vivo.

### Effects of puerarin on heart weight index

In this study, there were no significant differences in body weights among the six groups (Fig. 1F). Figure 1G shows that ISO damaged rats exhibited marked augmentation of HWI (*p* < 0.001) compared to control rats, which also indicated that the ISO-induced MI method was reliable. A significant decreasing trend in HWI was observed in the Pue-L and Pue-H groups compared to the ISO-damaged group (*p* < 0.001), while an altered trend was also observed in the Pue-Y group (*p* < 0.01). The results demonstrated that the new crystal form of puerarin could clearly relieve cardiac hypertrophy, and the improvement were better than that of the original crystal form of puerarin.

### Effects of puerarin on cardiac marker enzymatic activity in serum

**Figure 2:**
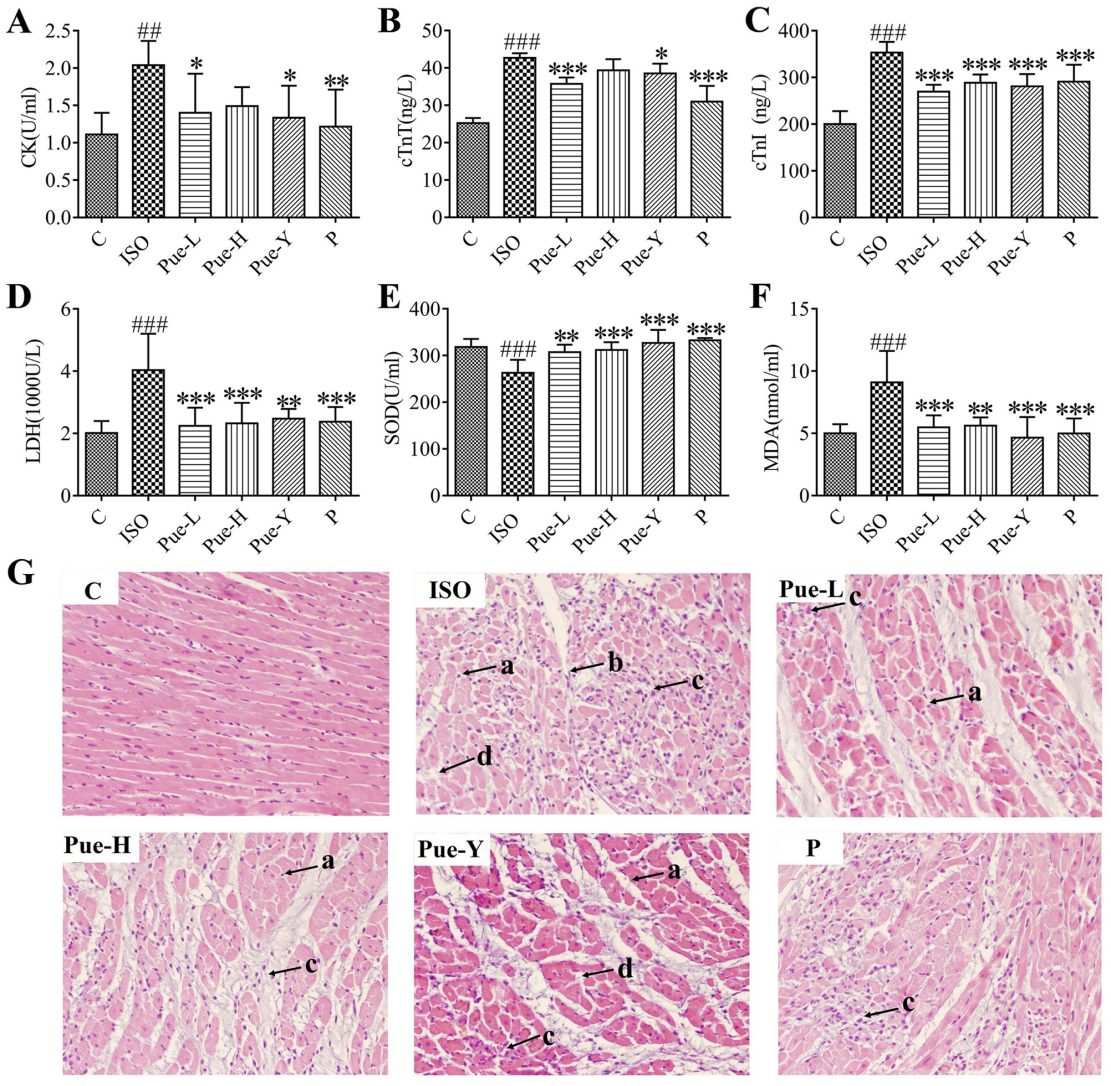
Effects of puerarin on myocardial enzymes and oxidative stress in serum (n = 6) and histopathological changes in heart tissues. (**A–D**) The contents of serum CK, cTnT, cTnI, LDH in rats of each group, respectively. (**E**,**F**) The levels of SOD and MDA in serum. Data are shown as Mean ± SD, (n = 6). ##*p* < 0.01and ###*p* < 0.001 vs control group; *p < 0.05, **p < 0.01, and***p < 0.001 *vs* ISO group. (**G**) Histopathological changes in heart tissues after ISO induced MI with hematoxylin–eosin (HE) staining (× 200 magnification). Black arrows refer to ISO-induced areas in the left ventricle. a. myocardial cell necrosis, b. disrupted myocardial fiber, c. inflammatory cell infiltration, d. edema.

CK, LDH, cTnT and cTnI have been used as representative biomarkers for the clinical diagnosis of MI. As shown in Fig. 2A–D, the levels of the four aforesaid indices in serum were notably elevated in the ISO-damaged rats compared with the levels in the control rats (*p* < 0.01). Further inspection showed that the CK levels in the Pue-L (30 mg/kg) and Pue-Y (120 mg/kg) groups decreased (*p* < 0.05) compared with those in the ISO group, and the activities of cTnT (*p* < 0.05), LDH (*p* < 0.01) and cTnI (*p* < 0.001) in the Pue-Y (120 mg/kg) group and Pue-L (30 mg/kg) group clearly decreased (*p* < 0.001). The results showed that the new crystal form of puerarin (30 mg/kg) could be effective in improving the biomarkers of MI, and the improvement effects were better than those of the original crystal form.

### Effects of puerarin on the oxidative stress indices in serum

There is a good amount of evidence suggesting that oxidative stress is strongly associated with the MI process^10,28^. As illustrated in Fig. 2E,F, the enzymatic activities of SOD decreased significantly (*p* < 0.001) and the contents of MDA significantly increased (*p* < 0.001) in the ISO group compared with the control group, while all the drug treatment groups significantly improved thses trends. Specifically, the alteration in MDA was significantly improved (*p* < 0.001) in the Pue-L (30 mg/kg) and Pue-Y (120 mg/kg) groups, and the activity of SOD was significantly (*p* < 0.001) reversed by the Pue-H (120 mg/kg) and Pue-Y (120 mg/kg) groups. These results indicated that the new crystal form of puerarin (30 mg/kg) could improve oxidative stress, and the abovementioned improvements of the new crystal form (30 mg/kg) were similar to the original crystal form (120 mg/kg) of puerarin.

### Effects of puerarin on histopathological analysis

Figure 2G presents the myocardial histological section results from the different groups. In the control group, the myocardial cells were arranged in an orderly fashion and exhibited clear nuclei. By contrast, apparent pathological changes occurred in the myocardial tissues of the model rats: there were obvious myocardial cell necrosis, local collagen fiber hyperplasia inflammatory cell infiltration, and interstitial edema of the myocardial tissues. Moreover, the infracted cardiac muscle exhibited a large number of structurally disordered fibrous tissues. In addition, all drug treatment groups showed amelioration of the ISO-induced injury to the myocardial tissues. Treatment with the new crystal form of puerarin showed cardioprotective effects without disruption of the myocardial fibers or obvious edema, indicating that the new crystal form could protect the myocardium from ischemic injury to some extent.

### Analysis of the ^1^H NMR profile

Figures S1A and S2A illustrate the typical ^1^H NMR spectra of the serum and cardiac tissues in the six groups, showing subtle changes in the levels of some vital endogenous metabolites. In the present study, Figs. S1B and S2B show the ^1^H NMR spectra for serum samples and heart samples from control rats. The major metabolites in the integrated regions assigned NMR signals were identified as specific metabolites based on literature data, the HMDB (https://www.hmdb.ca/) and the BMRB (https://www.bmrb.wisc.edu/); the numbered peaks of the metabolites in serum shown in Fig. S1B correspond to Table S1, and the numbered peaks of the metabolites in cardiac tissue shown in Fig. S2B correspond to Table S2.

### Multivariate data analysis

**Figure 3:**
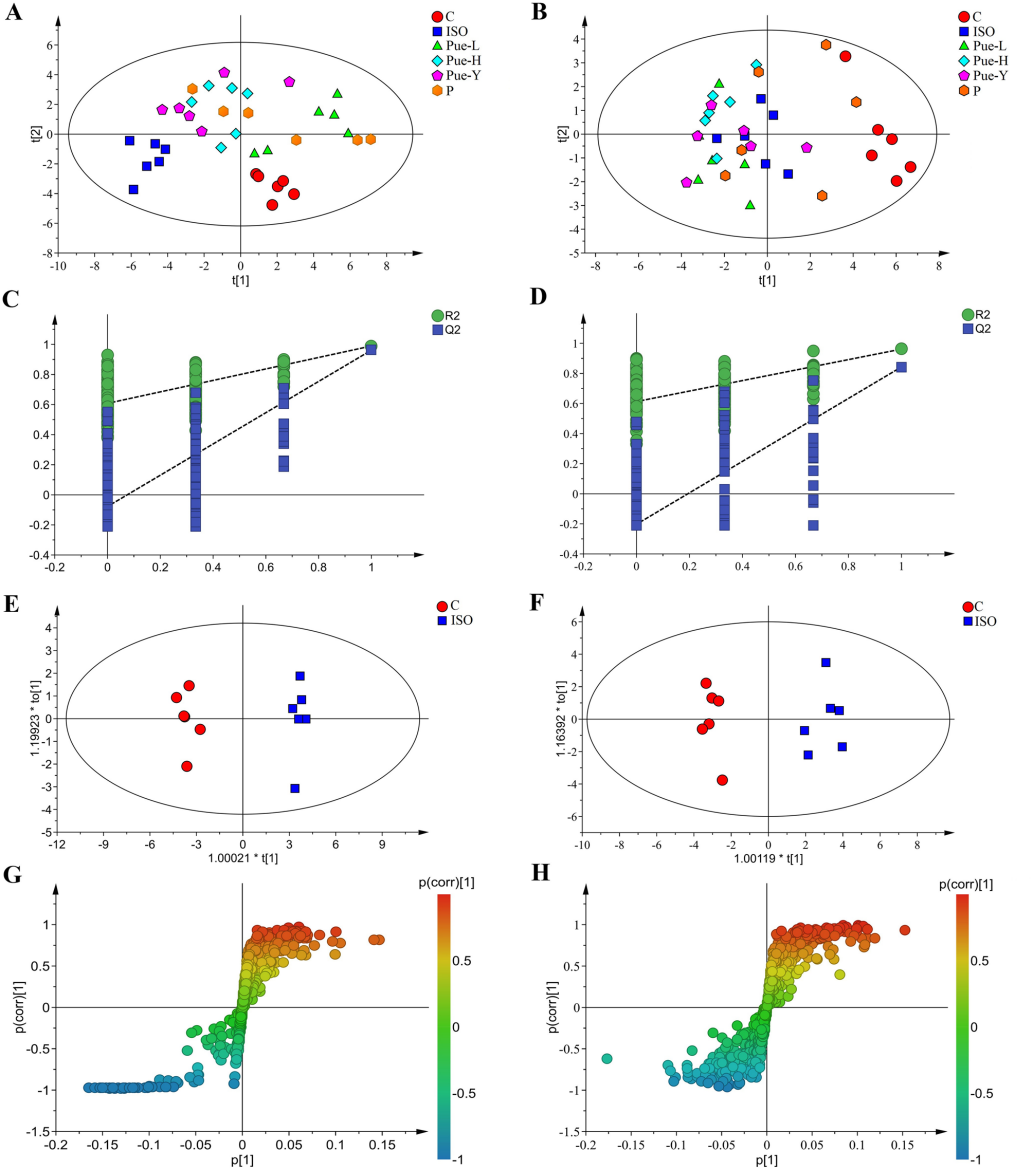
The metabolic profiles of serum and heart samples. The PCA score plot in serum (**A**) and in cardiac tissues (**B**) among the six groups. The PLS-DA validation plot obtained from 200 permutation tests in serum (**C**) and in cardiac tissues (**D**) for the models of normal and model groups. The OPLS-DA score plot in serum (**E**) and in cardiac tissues (**F**) from the control and ISO groups. The S-plots of OPLS-DA score plot in serum (**G**) and in cardiac tissues (**H**) between the control and ISO groups.

To obtain more detailed metabolic differences for the different samples among the six groups, all NMR data were subjected to multivariate data analysis. PCA was first performed for the whole data set to explore the potential outliners of the metabolite profile and to view the clustering trend of the six different experimental groups. The PCA score plots in serum (Fig. 3A) and heart samples (Fig. 3B) both showed satisfactory classification results with an explicit separation between the control group and ISO-damaged group, indicating a successful modeling process and an extremely severe metabolic disturbance in the ISO-damaged rats.

In the present study, the statistical validity of the control and ISO groups was assessed by permutation tests with cross validation, as shown in Fig. 3C,D. Parameter R2 explains the variance, Q2 represents the predictive ability of the model validation, and the degree of overlap between the permuted and original models accounts for the correlation. Both parameters provide a measure of model quality. The good predictability of the models was suggested by a negative Q^2^ intercept. The R^2^ and Q^2^ test results of the serum were 0.608 and −0.078, while the R^2^ and Q^2^ test results of the cardiac tissues were 0.613 and -0.203, respectively, indicating that the models reliably explained and predicted the variation.

Furthermore, supervised OPLS-DA was used to discriminate between the classes and identify the metabolites that distinguished the control group from the ISO group, and a significant division was observed between the two groups in both serum and heart samples (Fig. 3E,F). In addition, the OPLS-DA models in serum (R^2^X = 0.644, Q^2^ = 0.961) and cardiac tissues (R^2^X = 0.592, Q^2^ = 0.853) presented good stability and prediction when the parameters R^2^X and Q^2^ were greater than 0.5. These results indicated significant differences in metabolite profiles between the control and model groups in both serum and cardiac tissues.

Potential biomarkers were extracted from the S-plot based on their contribution to the differences, and the metabolites close to the two tails were suggested to have a good influence. The OPLS-DA S-plots (Fig. 3G,H) revealed the different metabolites between the control and model groups in serum and heart samples, respectively. In the S-plots, different points signify different metabolites; the farther away from the center of the variable, the better the influence of the variable on the separation of the groups.

### Analysis of the biomarkers in different samples

Metabolic analysis was used to explore important compounds and metabolic pathways related to MI in our study. Potential biomarkers were extracted from the S-plot based on their contribution to the differences, and the further the variable deviated from the origin, the higher the VIP value. Furthermore, the VIP values of each metabolic feature were also calculated. We set a significance threshold greater than 1.0 to select influential metabolites, and each VIP was ranked according to its contribution to the classification. Independent t-tests of relative peak area of the metabolites between the control and model groups was used to calculate the *p*-values (*p* < 0.05). As a result, based on the *p*-values (*p* < 0.05) and VIP values (VIP > 1), the biomarkers were identified.

**Figure 4:**
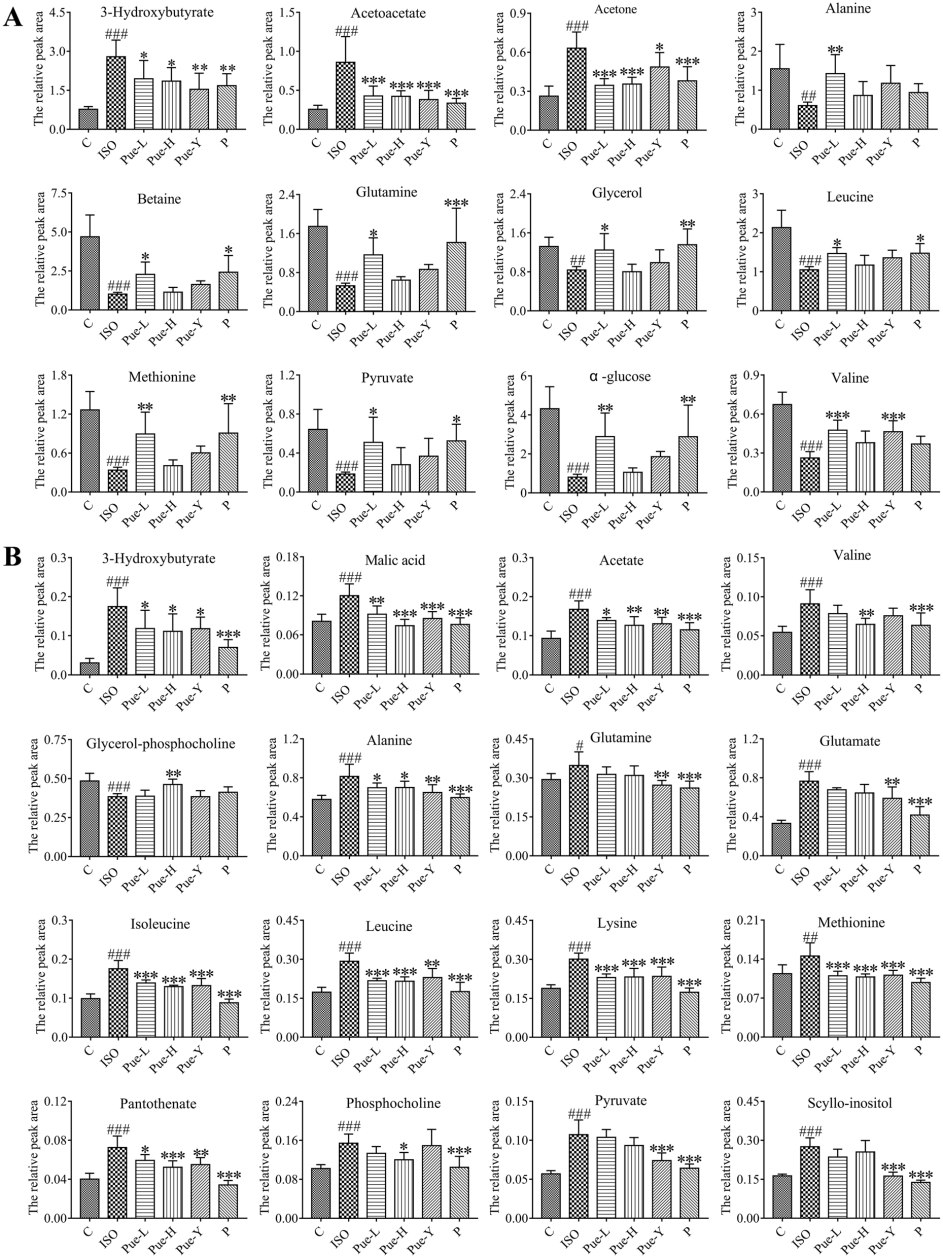
Relative peak areas of the potential biomarkers regulated by puerarin. (**A**) metabolites in serum. (**B**) metabolites in heart tissues. #*p* < 0.05, ##*p* < 0.01 and ###*p* < 0.001 vs control group; *p < 0.05, **p < 0.01 and***p < 0.001 vs ISO group.

In serum, 20 metabolites were identified as potential biomarkers, including organic acids (3-hydroxybutyrate, acetone, acetoacetate, and acetate), amino acids (isoleucine, leucine, valine, glutamate, glutamine, glycine, methionine, and alanine), α-glucose, pyruvate, lipids, choline, O-acetyl-glycoproteins (OAGs), glycerol, trimethylamine N-oxide (TMAO) and betaine. Furthermore, the results showed that 3 metabolites (3-hydroxybutyrate, acetone and acetoacetate) were obviously elevated in ISO-damaged rats, together with a notable reduction in the other biomarkers compared with the control group. Detailed information about the metabolites significantly regulated by the different crystal forms of puerarin in serum is shown in Fig. 4A. Further observation showed that the expression of 4 potential metabolites from the serum samples were reversed by the original crystal form of puerarin (120 mg/kg), while treatment with the new crystal form (30 mg/kg) could effectively inhibit the abnormalities in these 12 potential metabolites. These results showed that the new crystal form of puerarin could improve the expression of more metabolites than the original crystal form, indicating that the new crystal form could effectively ameliorate disturbance in the metabolites in ISO-induced MI rats.

In cardiac tissues, 22 metabolites were identified as potential biomarkers, including betaine, alanine, leucine, glutamine, phosphocholine, methionine, α-glucose, hypoxanthine, lactate, scyllo-inositol, aspartate, pyruvate, glutamate, acetate, lysine, dimethylglycine, malic acid, valine, 3-hydroxybutyrate (3-HB), pantothenate, isoleucine, and glycerophosphocholine (GPC). Furthermore, the results showed that 5 metabolites (GPC, betaine, hypoxanthine, lactate, and α-glucose) clearly decreased in ISO-damaged rats, together with a notable elevation in the other biomarkers compared with the control group. The relative peak areas of the metabolites in cardiac tissues significantly regulated by puerarin are shown in Fig. 4B. Further observations showed that the new crystal form (30 mg/kg) could effectively inhibit the abnormalities of the 9 metabolites, and the new crystal form (120 mg/kg) could significantly improve 12 altered metabolites. These results indicated that the different dosage of the new crystal form of puerarin was effective in treating MI. Moreover, the new crystal form of puerarin (120 mg/kg) was better than the original crystal form (120 mg/kg) in adjusting the intensity of metabolites such as valine, leucine, malic acid, acetate, isoleucine, GPC, phosphocholine, pantothenate, lysine, and methionine.

### Metabolic pathway analysis

**Figure 5:**
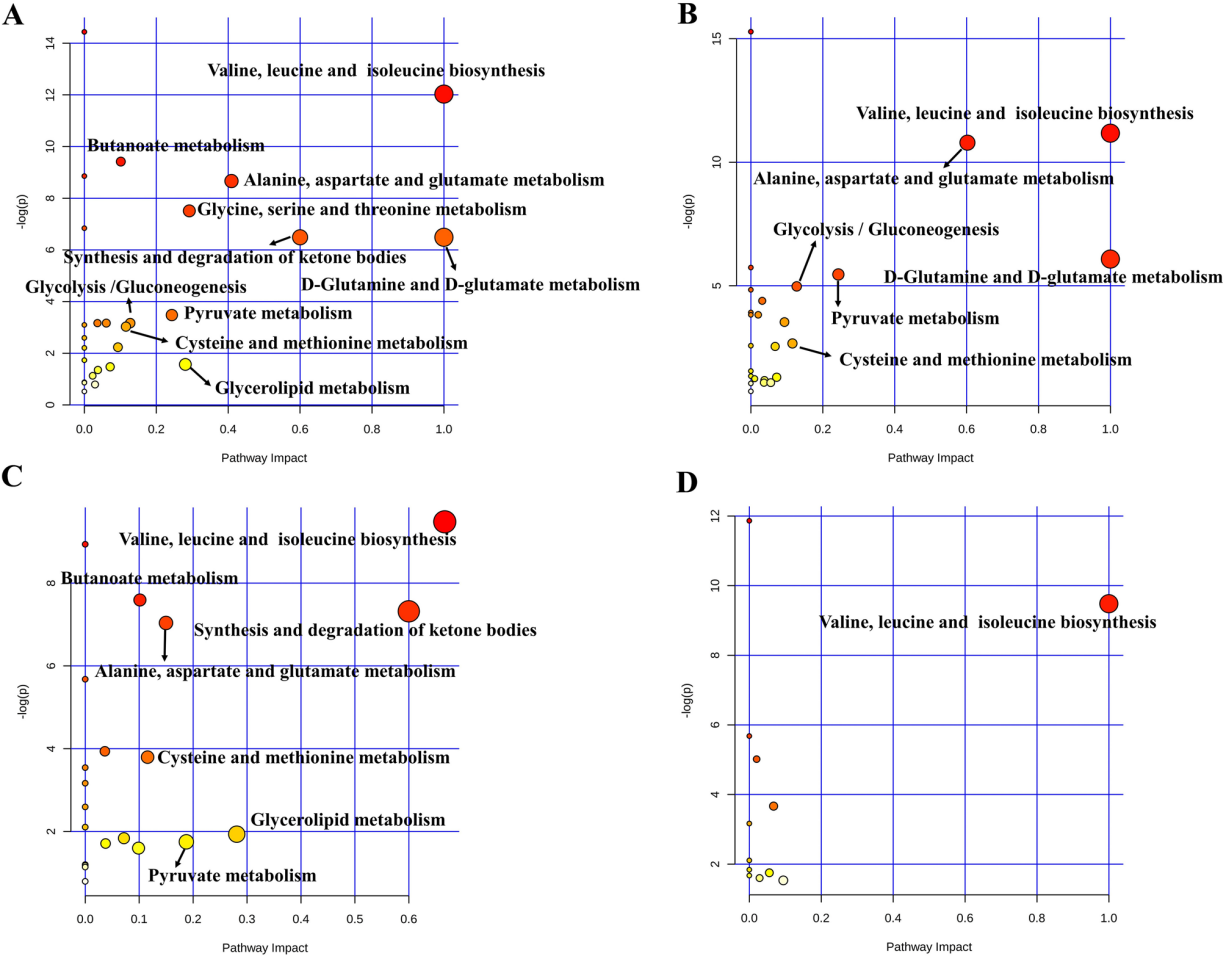
Summary of pathways analysis. (**A**) 10 disorder metabolic pathways in serum in the model of MI. (**B**) 6 metabolic pathways in cardiac tissues in the model of MI. (**C**) 7 metabolic pathways in serum and (**D**) 1 altered metabolic pathways in cardiac tissues regulated by the new crystal form of puerarin. The size and color of each circle indicate the significance of pathway ranked by *p* value and pathway impact score, respectively. (red represented higher *p* values and yellow represented lower *p* values, the larger the circle, the higher the impact score).

To recognize the metabolic pathways that are the most relevant to myocardial damage, MetaboAnalyst was used to integrate all potential biomarkers. Pathways with impact values > 0.1 were considered the most relevant pathways in this study. The results showed that a total of 10 metabolic pathways in serum (Fig. 5A) and a total of 6 metabolic pathways in cardiac tissue (Fig. 5B) were disordered in the MI model. Then, in order to illustrate the cardioprotective mechanisms of the new crystal form of puerarin, metabolic pathway analysis was performed by integrating the metabolites regulated by the new crystal form of puerarin. The results showed that the new crystal form of puerarin was effective in regulating the 7 metabolic pathways in serum (Fig. 5C) and valine, leucine and isoleucine biosynthesis in cardiac tissue (Fig. 5D). These results indicated that the new crystal form of puerarin was effective in treating myocardial injury and had a great influence on the regulation of the metabolic pathways in serum. The abovementioned metabolism mainly involved energy metabolism and amino acid metabolism.

### Heatmap analysis of the metabolites altered by the new crystal form of puerarin in serum and heart tissue

**Figure 6:**
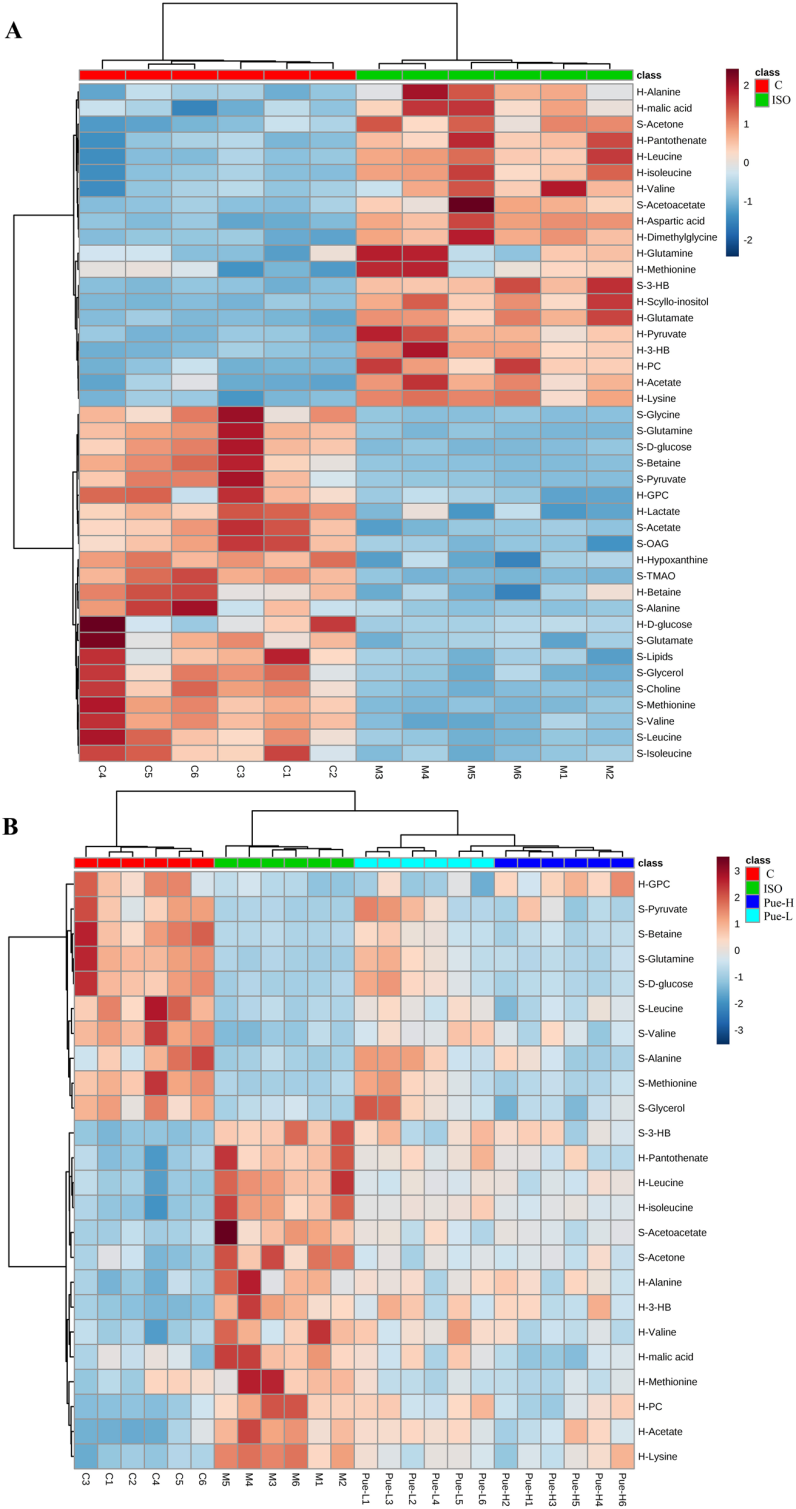
The heatmap analysis of altered metabolites in serum and heart tissues. (**A**) The heatmap analysis of the 42 metabolites in control and ISO groups and (**B**) the 24 puerarin-regulated metabolites in Control, ISO, Pue-L and Pue-H groups. And S represented the levels of metabolites in the serum, and H represented that in heart.

In this study, 20 metabolites from serum and 22 metabolites from cardiac tissue were identified as potential biomarkers for MI. In order to illustrate the relationships of these endogenous metabolites in MI, clustering analysis was performed. Heatmaps (Fig. 6A) present the changing trends of all 42 metabolites in the serum and heart samples between the control and model groups. Red represents an increase in the metabolite, and blue represents a decrease compared to the mean of the relative peak area. The analysis results displayed satisfactory clustering, and individuals in different groups were assembled together according to the metabolite change trends. Compared with the control group the levels of 20 metabolites increased and 22 metabolites decreased in the ISO-induced group. These results further demonstrated that construction of the MI model was successful and that the levels of endogenous metabolites were fully disordered in MI.

Furthermore, a total of 24 metabolites in serum and cardiac tissue were regulated by the new crystal form of puerarin in this study. Heatmap analysis of these metabolites was performed among the control, ISO, Pue-L and Pue-H groups (Fig. 6B). The analysis results displayed satisfactory clustering, and individuals in each group assembled together according to the change trends of the metabolites. The new crystal form of puerarin could significantly reverse the change trends of metabolites, such as 3-HB, leucine, isoleucine, acetoacetate, methionine, valine, alanine, pyruvate, alanine, glucose, and glutamine in the MI model. These metabolites were mainly involved in energy metabolism and amino acid metabolism.

### Correlation analysis of metabolites altered by the new crystal form of puerarin in serum and heart tissues

**Figure 7:**
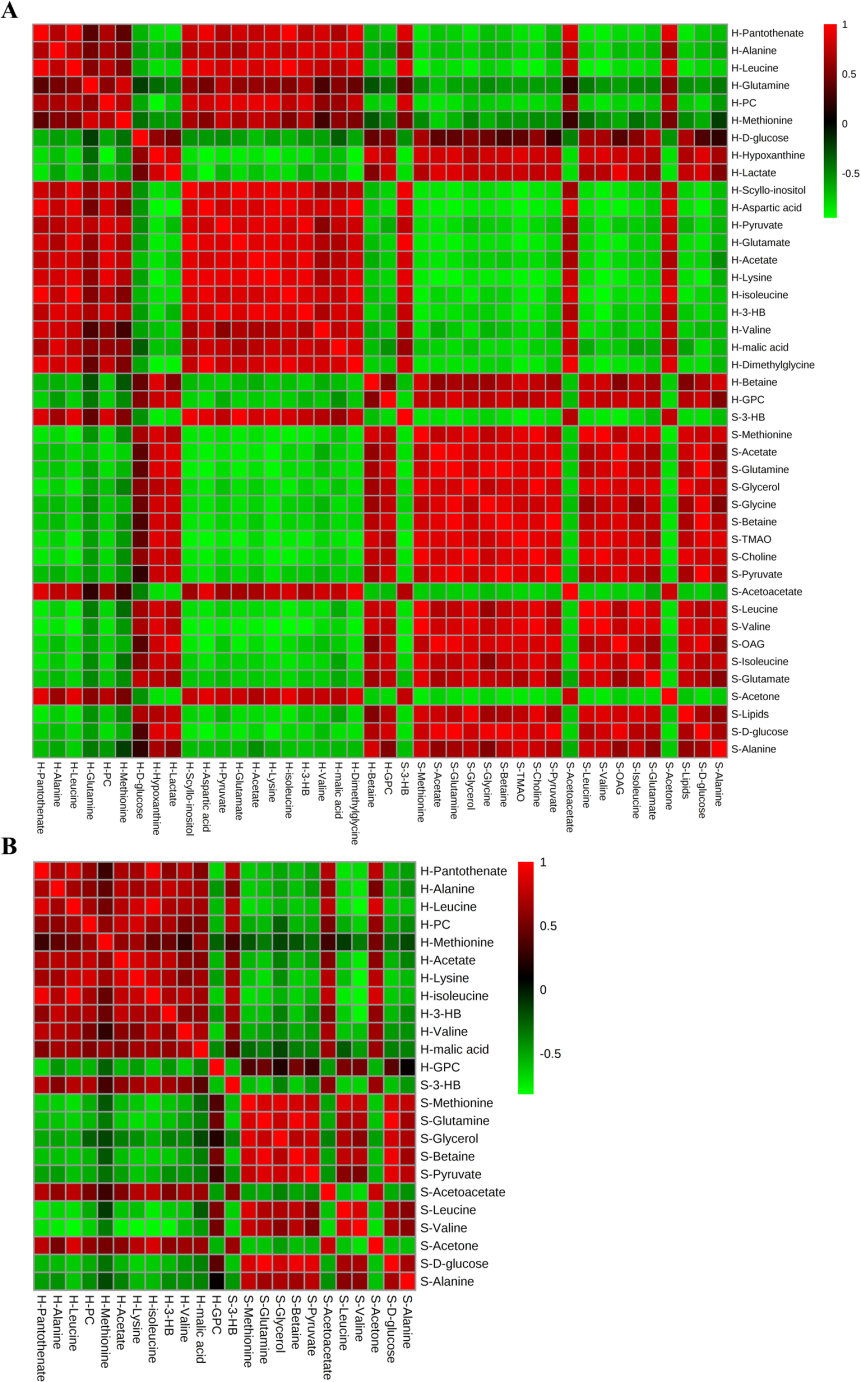
Correlation analysis of altered metabolites in serum and heart tissues. (**A**) The correlation heatmap analysis of 42 metabolites in MI. (**B**) The correlation heatmap analysis of 24 puerarin-regulated metabolites in MI.

In addition, in order to explore potential correlations between MI-related metabolites, correlation heatmap analysis was performed (Fig. 7A). As shown in Fig. 7A, based on the Pearson correlation coefficients, the positive and negative correlations between metabolites are presented in the plot with red and green, respectively. The results showed that all amino acids in myocardial tissues (alanine, leucine, methionine, aspartate, glutamate, lysine, isoleucine, valine) were negatively correlated with the amino acids in serum (isoleucine, leucine, valine, glutamate, glutamine, glycine, methionine, alanine), indicating that in MI, the amino acid change trends were the opposite between the two kinds of analysis samples. Moreover, the energy metabolism-related metabolites in the heart samples (α-glucose, hypoxanthine, and lactate) had similar change trends with the same type of metabolites in serum (α-glucose and pyruvate), and these metabolites were distributed in the red clusters. In addition, in serum, the key metabolites of pyruvate metabolism (pyruvate and acetate) and glycerolipid metabolism (glycerol) were positively correlated with the different amino acids, while the key metabolites of the synthesis and degradation of ketone bodies (3-HB and acetoacetate) were negatively correlated with the amino acids. In myocardial tissue, the key metabolites of pyruvate metabolism (pyruvate and acetate) were positively correlated with the different amino acids. The results showed that there were relationships among the different metabolites.

In order to explore the potential correlations of 24 metabolites in serum and cardiac tissue regulated by the new crystal form of puerarin in this study and to illustrate the potential mechanism, correlation heatmap analysis was performed (Fig. 7B). The results showed that the 6 amino acids in the heart samples (alanine, leucine, methionine, lysine, valine and isoleucine) were negatively correlated with the 4 amino acids in serum (leucine, valine, methionine, and alanine), indicating that the amino acid change trends regulated by the new crystal form of puerarin were the opposite between the two kinds of analysis samples. In addition, as the key metabolites of valine, leucine and isoleucine biosynthesis, leucine and valine in serum were negatively correlated with the key metabolites of the synthesis and degradation of ketone bodies (3-HB and acetoacetate), indicating that the change trends of these metabolites were the opposite between the two kinds of metabolism. However, leucine and valine in serum were positively correlated with the key metabolites of the metabolism of other amino acids in serum, including methionine in cysteine and methionine metabolism and alanine in alanine, aspartate and glutamate metabolism. Moreover, leucine and valine in serum had similar change trends with pyruvate in pyruvate metabolism and glycerol in glycerolipid metabolism, and these amino acids were distributed in the red clusters. In addition, in myocardial tissues, valine, leucine and isoleucine were positively correlated with the amino acids, lysine, alanine and methionine. The above results showed that there were relationships among the different metabolites regulated by the new crystal form of puerarin. These metabolites were mainly involved in amino acid metabolism and energy metabolism, further indicating that the new crystal form is mainly involved in regulating these types of metabolism to exert cardioprotective effects.

## Discussion

Myocardial ischemia causes physiological, pathological and morphological changes to myocardial tissues, leading to abnormal heart functions and abnormal structures. Previous research has shown that ISO administration is widely used to establish a model of MI^29–31^. In this study, the cardioprotective effects of the new crystal form of puerarin in ISO-damaged experimental rats was evaluated by electrocardiogram, echocardiography detection, biochemical assays at the serum level, myocardial histology analysis, and untargeted metabolomics based on NMR analysis both in serum and heart tissues. The results confirmed that the new crystal form of puerarin had significant protection against ISO-induced MI. The protective effects of the new crystal form of puerarin at a low dose (30 mg/kg) was equivalent to or better than that of the original crystal form at a high dose (120 mg/kg) in some indices. The results suggested that changing the crystal form of puerarin was an effective approach to improve the treatment efficacy, which could serve as a reference for the pharmaceutical crystallography applications for poorly soluble TCM components.

**Figure 8:**
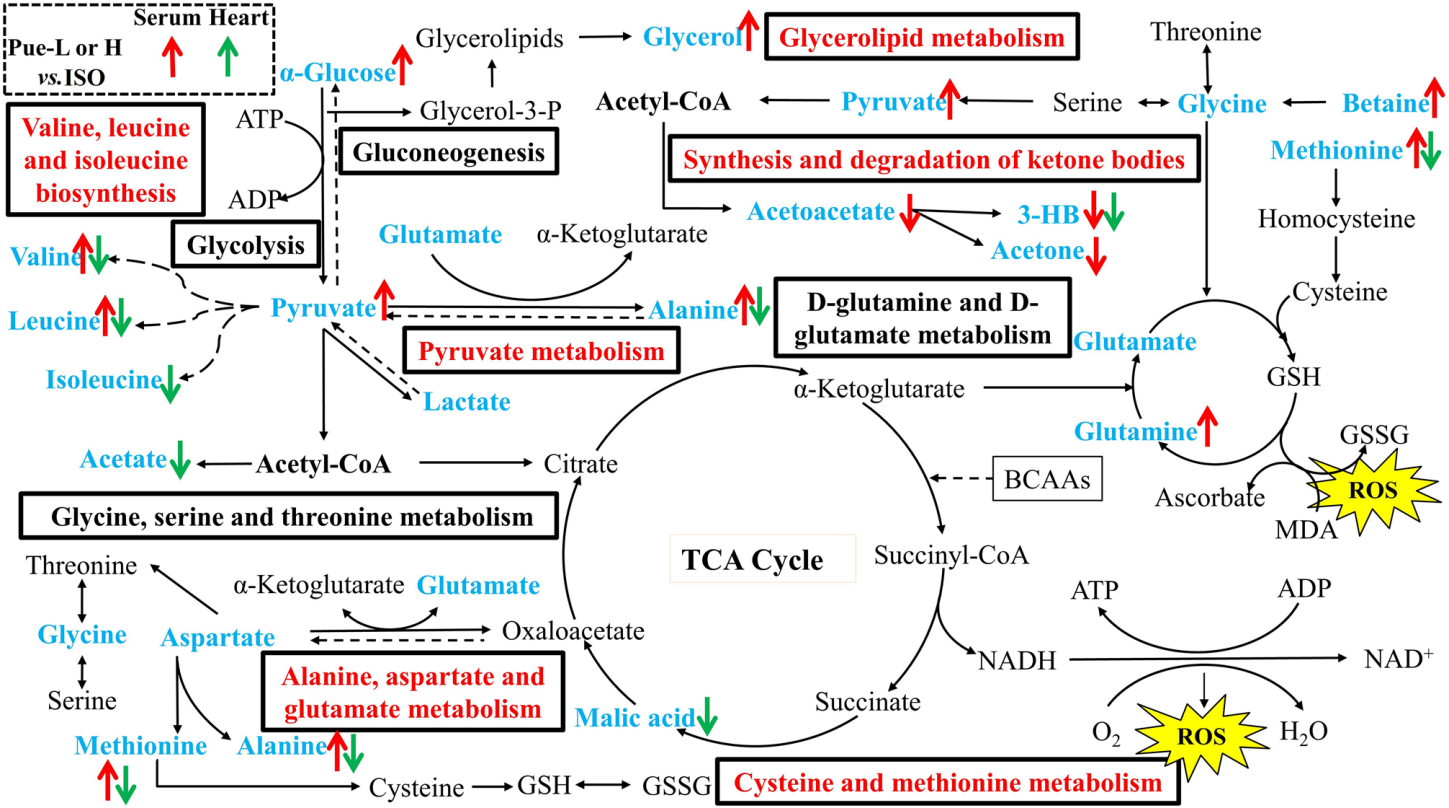
Alterations of metabolites and possible metabolic pathways in myocardial tissues and serum. The metabolites in blue font were altered in MI and metabolic pathways in red font were regulated by the new crystal form of puerarin. And the different colors of arrows represent the altered trends of metabolites that were regulated by the new crystal form of puerarin in the serum and myocardial tissues (red represented the serum, and green represented the myocardial tissues) in the Pue-L or Pue-H groups compared with the model groups, respectively.

More specifically, in our study, ISO caused a significant increase in the heart weight index and abnormal myocardial parameters, such as EF (%) and FS (%), which were in accordance with the disease symptoms of MI and indicated that the model was validated. The aforementioned indices could be further regulated by the new crystal form of puerarin, and its trends in improvement were superior to those of the original crystal form of puerarin at the same dose. In the serum biochemical assays, treatment with the new crystal form of puerarin could significantly suppress oxidative stress, including an increase in the activity of SOD and a decrease in the level of MDA. On the other hand, the levels of CK, LDH, cTnI, and cTnT significantly increased in the serum of the ISO-damaged rats, and rats pretreated with the new crystal form of puerarin exhibited improved trends in these indices. In addition, metabolomics based on ^1^H-NMR analysis was performed to discover metabolic alterations in serum and cardiac tissues. The results showed that some endogenous metabolites changed, and the new crystal form of puerarin could ameliorate the metabolic disturbance, which was mainly associated with amino acid metabolism, oxidative stress and energy metabolism. The metabolites regulated by the new crystal form of puerarin and the relevant metabolic pathways were shown in Fig. 8.

### Amino acid metabolism

There is a great demand for energy in cardiac tissues. Blood flow to the heart decreases when there is a partial blockage of tissues, leading to reduced oxygen consumption and reduced adenosine triphosphate (ATP) production^32^. Certain amino acids, regarded as cardioprotective substrates, are converted to metabolic intermediates during ischemia to increase the supply of ATP. In the present study, a wide range of amino acids (isoleucine, leucine, valine, glutamate, alanine, methionine, glycine, lysine, and aspartate) were altered in cardiac tissues and serum samples. These metabolites were primarily involved in amino acid metabolism, including D-glutamine and D-glutamate metabolism; valine, leucine and isoleucine biosynthesis; alanine, aspartate and glutamate metabolism; glycine, serine and threonine metabolism; and cysteine and methionine metabolism, which also demonstrates that changes in amino acid metabolism play important role in the progression of MI. The possible reason for this result is that the energy requirement in the myocardium could be met by the consumption and biosynthesis of amino acids. Some amino acids, such as glutamate, glutamine, and aspartate, and branched chain amino acids (BCAAs, valine, leucine and isoleucine), are preferentially used as metabolic substrates in the tricarboxylic acid cycle (TCA cycle)^33^. In this study, the metabolites (alanine, glutamine, isoleucine, valine, leucine, methionine, and lysine) in serum and myocardial tissue were regulated by the new crystal form of puerarin and were mainly involved in follow amino acid metabolism to exert cardioprotective effects: valine, leucine and isoleucine biosynthesis; alanine, aspartate and glutamate metabolism; and cysteine and methionine metabolism.

### Valine, leucine and isoleucine biosynthesis

It is well known that BCAAs play crucial roles in energy production as alternative energy substrates^34^. Myocardial tissues have relatively high capacities to degrade and utilize BCAAs compared with other tissues^35^. A previous study showed that BCAAs accumulation in cardiac tissues was a metabolic signature of heart failure models^36^. Sun et al. found that BCAAs catabolic defects resulted in the accumulation of branched chain α-keto acids, which caused reactive oxygen species (ROS) injury in cardiac tissues and significantly contributed to the progression of heart failure^37^. Previous literature has also reported that the accumulation of BCAAs in myocardial tissues leads to mitochondrial dysfunction, energy metabolism disturbance and mammalian target of rapamycin (mTOR) signaling pathway activation, which further exacerbates cardiac dysfunction and remodeling^36,38^. In this study, the levels of leucine, isoleucine and valine in myocardial tissues were significantly increased in the MI model. These results were in accordance with previous literature studies. Treatment with the new crystal form of puerarin significantly reversed these change trends. In contrast, Guo et al. found that ISO treatment resulted in a significant reduction in amino acids (such as leucine and valine) in plasma, and treatment with Danshen dripping pills could effectively relieve MI induced by ISO^39^. In this study, the levels of leucine and valine were decreased in serum. Treatment with the new crystal form of puerarin effectively reversed their levels in serum. These results were in accordance with previous literature reports. The BCAA contents in different analysis samples exhibited different trends in this study, and the heatmap analysis and correlation analysis also demonstrated these results. Previous literature has also reported that some metabolites showed different tendencies in two kinds of analysis samples after ISO induction in the MI model^39–41^. The possible reason for this result was that serum samples reflect physiological and biochemical changes in the entire body of rats, which is much more complicated than a simple tissue.

### Alanine, aspartate and glutamate metabolism

Alanine, glutamate and aspartate were the abundant free amino acids, and were associated with energy metabolism as preferential fuel. A previous study showed that glutamate content was decreased in serum in MI accompanied by pyruvate deficiency, and the latter contributed as substrates or intermediates in the processes of glycolysis and the TCA cycle for further ATP production^18^. Aquilani et al. found that arterial amino acid levels (such as aspartate, glutamate, methionine and cysteine) were reduced to make up for the metabolic needs of patients, and the levels of amino acids decreased with increasing disease severity in chronic heart failure patients^42^. In this study, the heatmap analysis (Fig. 6A) showed that alanine, glutamate and pyruvate in serum decreased in the model rats compared with normal rats. The new crystal form of puerarin could reverse the alanine and glutamate contents. Our results were consistent with those found in previous literature studies. In contrast, in myocardial tissues, previous studies have shown that significant quantities of glutamate accumulated in the hearts of rats with heart failure, which was related to the reduced activities of glutamate cyclase^43^ and the malfunction of glutamate transporters during ischemic injury^44^. In this study, as shown in Fig. 6, the levels of glutamate, alanine and aspartate were elevated in the model rats. Administration of the new crystal form of puerarin could effectively reverse the levels of glutamate and alanine. Our results were in accordance with the previous literature in myocardial tissues. Moreover, the correlation analysis of metabolites in this study (Fig. 7) also showed that alanine and glutamate in serum were negatively correlated with aspartate, alanine and glutamate content in myocardial tissues. The results also demonstrated that the altered trends in metabolites in the different analysis samples were different during MI.

### Cysteine and methionine metabolism

Cysteine and methionine metabolism is an amino acid metabolism that is closely related to oxidative stress. ROS are excessively generated in some pathophysiological conditions, such as inflammation or myocardial injury. Methionine is one of the most representative amino acids that contains the sulfhydryl group, and it is extremely sensitive to all forms of ROS^45^. In addition, methionine can effectively regulate metabolic processes and the immune system, activate the activities of endogenous antioxidant enzymes and synthesize glutathione to counteract oxidative stress^46^. A previous study demonstrated that methionine could alleviate oxidative stress induced by various oxidants and protect cardiac tissues and vasculature from damage^45^. In this study, the levels of methionine and the activities of SOD decreased in the serum of model rats, and the levels of MDA increased. It is possible that this reduction in methionine in serum resulted in aggravation of the damaging effects of oxidative stress. Administration of the new crystal form of puerarin could effectively reverse the situation in the serum and further improve cysteine and methionine metabolism. In contrast, in myocardial tissues, Chaturvedi et al. found that high levels of methionine could lead to high oxidative stress in the heart and cause cardiac threats^47^. The reason for this finding may be that methionine accumulation in myocardial tissues leads to increased levels of homocysteine and then gives rise to a condition called hyperhomocysteinemia, which is considered a cardiovascular risk^48^. In addition, Chaturvedi et al. found that excessive methionine in myocardial tissues affects cardiac function by increasing oxidative stress, inflammatory manifestations, and cardiac remodeling and decreasing cardiac function^47^. In this study, the methionine content in model rats increased in cardiac tissues compared with normal rats. Different dosages of the new crystal form of puerarin could effectively improve the levels of methionine. The results were in accordance with previous literature.

### Energy metabolism

The progression of MI was accompanied by inadequate energy metabolism. The TCA cycle plays a crucial role in producing ATP in the myocardium, and lipids, glucose, amino acids and ketone bodies are known to be the key energy sources that provide substrates for cardiac metabolism. In this study, except for amino acid metabolism, these energy-related metabolites were regulated by the new crystal form of puerarin and were involved in the following types of metabolism: synthesis and degradation of ketone bodies, glycerolipid metabolism, and pyruvate metabolism.

### Synthesis and degradation of ketone bodies

The synthesis and degradation of ketone bodies is one of the metabolic pathways related to fatty acid degradation. The synthesis of ketone bodies (3-HB, acetoacetate, and acetone) increases in the mitochondria of the liver when glucose levels are low. However, ketone bodies have no effect in hypoxic states since they require mitochondrial electron transport to exert their effects, leading to the accumulation of ketone bodies in serum and myocardial tissues^49^. In this study, compared with normal rats, ketone bodies in serum and myocardial tissues were increased in MI rats. The new crystal form of puerarin could effectively decrease 3-HB in myocardial tissues and 3-HB, acetoacetate, and acetone in serum to improve the synthesis and degradation of ketone bodies. Our results were in accordance with the previous literature.

### Glycerolipid metabolism

For myocardial ischemia, lipids can also produce energy by β-oxidation. Glycerolipid metabolism is one of the metabolic pathways related to fatty acid degradation. Glycerol plays important roles in cell energy generation and lipid synthesis. Wang et al. found that the levels of glycerol and lipids were disordered in the plasma in a model of chronic progressive heart failure, and significant changes in metabolites in plasma indicated that ischemia led to a lipid metabolism disorder via glycerolipid metabolism^50^. In this study, the levels of glycerol and lipids decreased in the serum of model rats, suggesting disturbances in glycerolipid metabolism. The disrupted level of glycerol was reversed by the new crystal form of puerarin (30 mg/kg), and the results indicated that the new crystal form of puerarin showed myocardial injury protective effects, which could improve energy metabolism.

### Pyruvate metabolism

Glycolysis is an important method of ATP production to meet energy demand. Pyruvate is the last glycolysis intermediate, and it can be further transferred to acetyl-coenzyme A (acetyl-CoA) in mitochondria; then, acetyl-CoA releases considerable energy through the TCA cycle^18^. Pyruvate metabolism in mitochondria is regulated by many enzymes to maintain a balance^51^. Guo et al. found that ISO induced a significant increase in pyruvate and a decrease in glucose in the heart tissues during MI, indicating that glycolysis in the heart was accelerated and the TCA cycle was inhibited^39^. In this study, heatmap analysis (Fig. 6) showed that α-glucose decreased and pyruvate increased significantly in myocardial tissues in MI rats compared with normal rats, indicating abnormal glycolysis and inhibition of the TCA cycle. The new crystal form of puerarin could effectively improve this situation. Our results were in accordance with the previous literature. However, pyruvate metabolism is complex in the body, and the levels of pyruvate may be upregulated or downregulated to treat disease in a compensatory or secondary manner^51^. A previous study showed that pyruvate was metabolized to lactate by LDH under anaerobic conditions during acute myocardial ischemia–reperfusion injury. This process could generate a small amount of ATP, which is required for glycolysis^52^. The possible reason for this result was that the glucose and pyruvate in serum were consumed to produce ATP and to meet the inadequate energy metabolism requirements^39^. In addition, Jiang et al. found that glutamate content decreased in the serum of MI rats accompanied by pyruvate deficiency^18^. In this study, the levels of glucose, pyruvate and glutamate in the serum of model rats decreased, and the new crystal form of puerarin could significantly reverse these altered trends.

### Oxidative stress

Previous studies showed that ISO administration to rats increased oxidative stress^4^. Impaired heart tissues have limited antioxidant capacity, making them particularly vulnerable to ROS and exacerbating oxidative damage^53^. Furthermore, oxidative damage results in lipid peroxidation, exacerbating cardiac dysfunction and remodeling. In this study, the metabolomics results showed that metabolites related to oxidative stress, such as glutamine, glutamate, methionine, valine, leucine, isoleucine and glycine, were obviously altered in model rats, while administration of the new crystal form of puerarin could markedly ameliorate the changes in glutamine, methionine, valine and leucine in the serum and the changes in glutamine, glutamate, methionine, valine, leucine, and isoleucine in myocardial tissues. In addition, in this study, the expression levels of SOD and MDA were altered in the serum of MI rats, indicating that ISO caused oxidative damage. Treatment with the new crystal form of puerarin could significantly suppress oxidative stress by modulating the production of lipid peroxides and augmenting the overall antioxidant defense system to protect cardiac tissues. Some research has shown that ROS generation causes lipid membrane disruption, resulting in the release of myocardial enzymes into the blood in rats with ISO-induced MI^4,54^. In this study, we also found that the levels of the enzymes CK, LDH, cTnI, and cTnT were significantly increased in the serum of ISO-damaged rats. Drug-pretreated rats exhibited different improvements in these indices. Above all, these findings may support that the new crystal form of puerarin could reverse the disturbances in oxidative stress.

In summary, in this study, untargeted metabolomics based on NMR analysis results showed that some metabolites exhibited different trends in serum and cardiac tissues, and the specific reasons still need to be explored. Last but not least, the potential mechanisms of the new crystal form of puerarin were involved in energy metabolism, while the myocardial contents of ATP and ADP in the present study were not examined to further confirm this conclusion.

## Conclusion

In this study, a combination of electrocardiogram, echocardiography detection and biochemical assays in serum were used to investigate the cardioprotective effects of a new crystal form of puerarin, and NMR analysis of both the serum and heart tissues was used to investigate its potential mechanisms. The new crystal form of puerarin was effective to treatment MI, and its cardioprotective effect was apparently superior to the original crystal form of puerarin. The potential mechanisms were related to the amino acids metabolism, oxidative stress and energy metabolism under MI conditions.

## Supplementary information

Supplementary Information.

## Abbreviations

MI: Myocardial ischemia
ISO: Isoproterenol
NMR: Nuclear magnetic resonance
SOD: Superoxide dismutase
MDA: Malonaldehyde
CK: Creatine kinase
cTnT: Cardiac troponin T
cTnI: Cardiac troponin I
LDH: Lactate dehydrogenase
EF %: Ejection fraction
FS %: Fractional shortening
TCM: Traditional Chinese Medicine
ATP: Adenosine-triphosphate
BCAAs: The branched chain amino acids
ROS: Reactive oxygen species
TCA cycle: Tricarboxylic acid cycle
