# Supplementary material for: The cardioprotective effects of the new crystal form of puerarin in isoproterenol-induced myocardial ischemia rats based on metabolomics
Source: Sci Rep. 2020 Oct 20;10:17787. doi: 10.1038/s41598-020-74246-y (PMC7575583; doi:10.1038/s41598-020-74246-y)
Supplement: Supplementary file 1 — Supplementary Information. [file 41598_2020_74246_MOESM1_ESM.docx]

**Supplementary information**

**The cardioprotective effects of the new crystal form of** [**puerarin**](https://www.sciencedirect.com/topics/medicine-and-dentistry/puerarin) **in isoproterenol-induced myocardial ischemia rats based on metabolomics**

**Yuzhi Zhou^1,2,3^, Mengru Li ^3^, Jia Song ^3^, Yongqiang Shi^2^, Xuemei Qin^3^, Zhaolin Gao^2^, Yang Lv^1^ & Guanhua Du ^1^***

^1^Institute of Materia Medica, Chinese Academy of Medical Sciences &Peking Union Medical College, 2A Nan Wei Road, Beijing 100050, China. ^2^Shandong Province Key Laboratory of Polymorph Drugs, Shandong Yikang Pharmaceutical Co.，Ltd., No.3288, Yikang Avenue, Tengzhou 277513, China. ^3^Modern Research Center for Traditional Chinese Medicine, Shanxi University, No.92, Wucheng Road, Taiyuan 030006, China. ^*^Corresponding author. Tel: 010-63165184.Key Laboratory of Drug Target Research and Drug Screen, Institute of Materia Medica, Chinese Academy of Medical Science and Peking Union Medical College, Beijing, 100050 China. E-mail: Du Guan-hua, [dugh@imm.ac.cn](mailto:dugh@imm.ac.cn)

**Supplementary Figures and Tables**

### Fig. S1. (A) Typical 600MHz ^1^H NMR spectra of serum samples from different groups. (B) ^1^H-NMR spectra of control rat for metabolites identified from the serum samples.

**Fig. S2.** (A) Typical 600 MHz 1H NMR spectra of cardiac tissues from different groups. (B) 1H-NMR spectra of control rat for metabolites identified from the cardiac tissues.

### Table S1. The list of the detailed NMR assignments from serum in rats.

### Table S2. The list of the detailed NMR assignments from cardiac samples in rats.


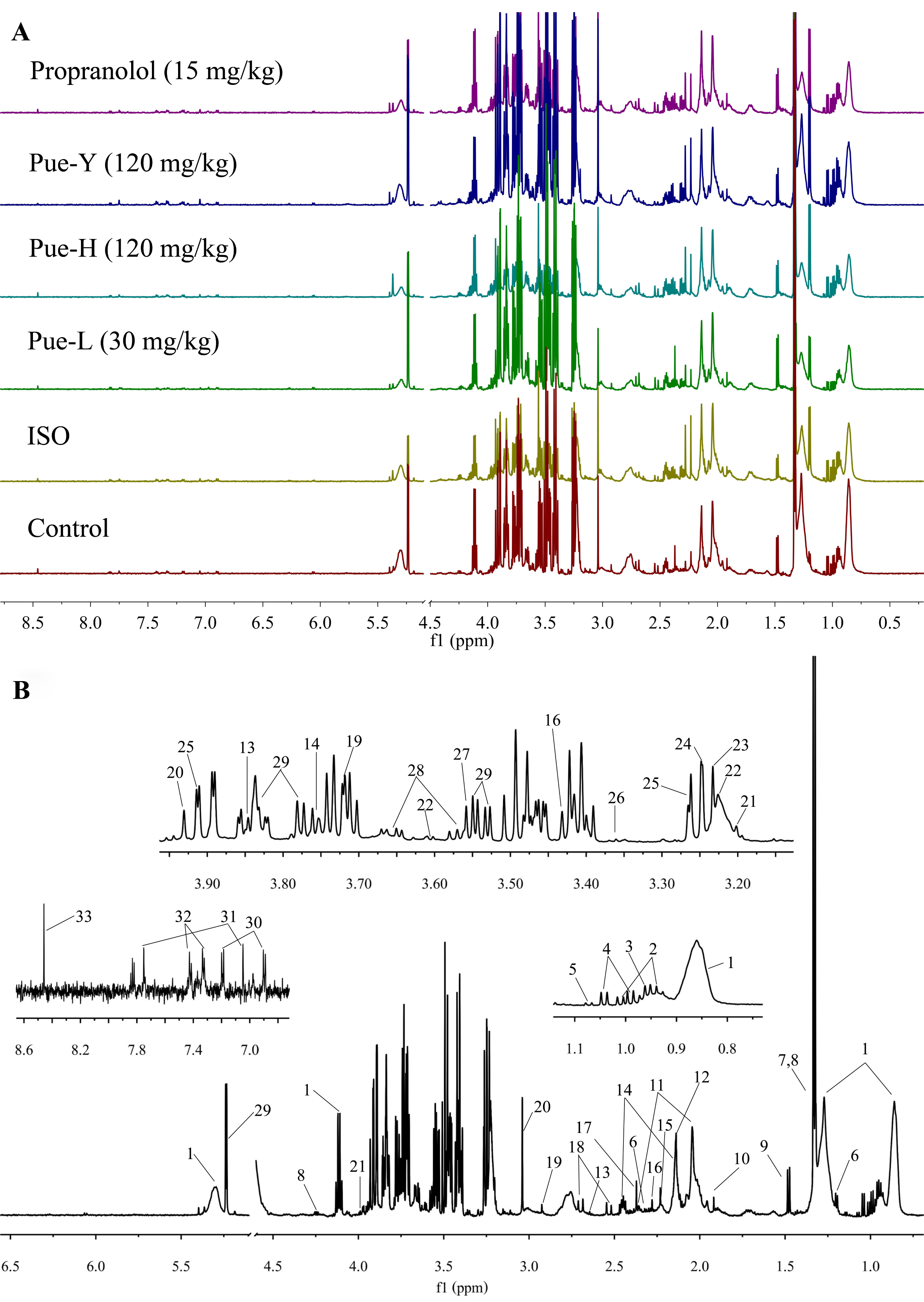


### Fig.S1. (A) Typical 600MHz ^1^H NMR spectra of serum samples from different groups. (B) ^1^H-NMR spectra of control rat for metabolites identified from the serum samples.

### Table S1. The list of the detailed NMR assignments from serum in rats

| NO. | Metabolites | δH( multiplicity^a^) | Samples |
| --- | --- | --- | --- |
| 1 | Lipids | 0.86(m),1.27(m),2.04(m), 5.30(m) | S |
| 2 | Isoleucine | 0.94(t),1.01(d) | S |
| 3 | Leucine | 0.96(t) | S |
| 4 | Valine | 0.99(d),1.04(d) | S |
| 5 | Isobutyric acid | 1.07(d) | S |
| 6 | 3-Hydroxybutyrate | 1.20(d),2.32(d),2.41(d) | S |
| 7 | Lactate | 1.33(d),4.11(q) | S |
| 8 | Threonine | 1.33(d),4.24(m) | S |
| 9 | Alanine | 1.48(d) | S |
| 10 | Acetate | 1.92(s) | S |
| 11 | Glutamate | 2.05(m),2.14(m),2.35(m) | S |
| 12 | O-Acetyl-glycoproteins | 2.14(s) | S |
| 13 | Methionine | 2.14(s),2.65(m),3.85(m) | S |
| 14 | Glutamine | 2.14(m),2.46(m),3.76(m) | S |
| 15 | Acetone | 2.23(s) | S |
| 16 | Acetoacetate | 2.28(s),3.43(s) | S |
| 17 | Pyruvate | 2.37(s) | S |
| 18 | Citrate | 2.53(d),2.70(d) | S |
| 19 | Dimethylglycine | 2.92(s),2.72(d) | S |
| 20 | Creatine | 3.04(s),3.93(s) | S |
| 21 | Choline | 3.20(s),4.06(m) | S |
| 22 | PC | 3.22(s),3.61(m) | S |
| 23 | GPC | 3.23(s) | S |
| 24 | TMAO | 3.25(s) | S |
| 25 | Betaine | 3.27(s) | S |
| 26 | Scyllo-inositol | 3.36(s) | S |
| 27 | Glycine | 3.56(s) | S |
| 28 | Glycerol | 3.59(m),3.66(dd) | S |
| 29 | a-Glucose | 5.24(d),3.54(dd),3.77(m),3.84(m) | S |
| 30 | Tyrosine | 6.90(d),7.19(d) | S |
| 31 | Histidine | 7.05(s),7.75(s) | S |
| 32 | L-Phenylalanine | 7.32(m),7.43(m) | S |
| 33 | Formate | 8.46(s) | S |

### Multiplicity^a^: singlet(s), doublet(d), triplet(t), doublet of doublets(dd), multiplet (m), quartets(q). TMAO: Trimethylamine N-oxide; PC: phosphocholine; GPC: glycerol-phosphocholine; OAG: O-Acetyl-glycoproteins; S: serum

**
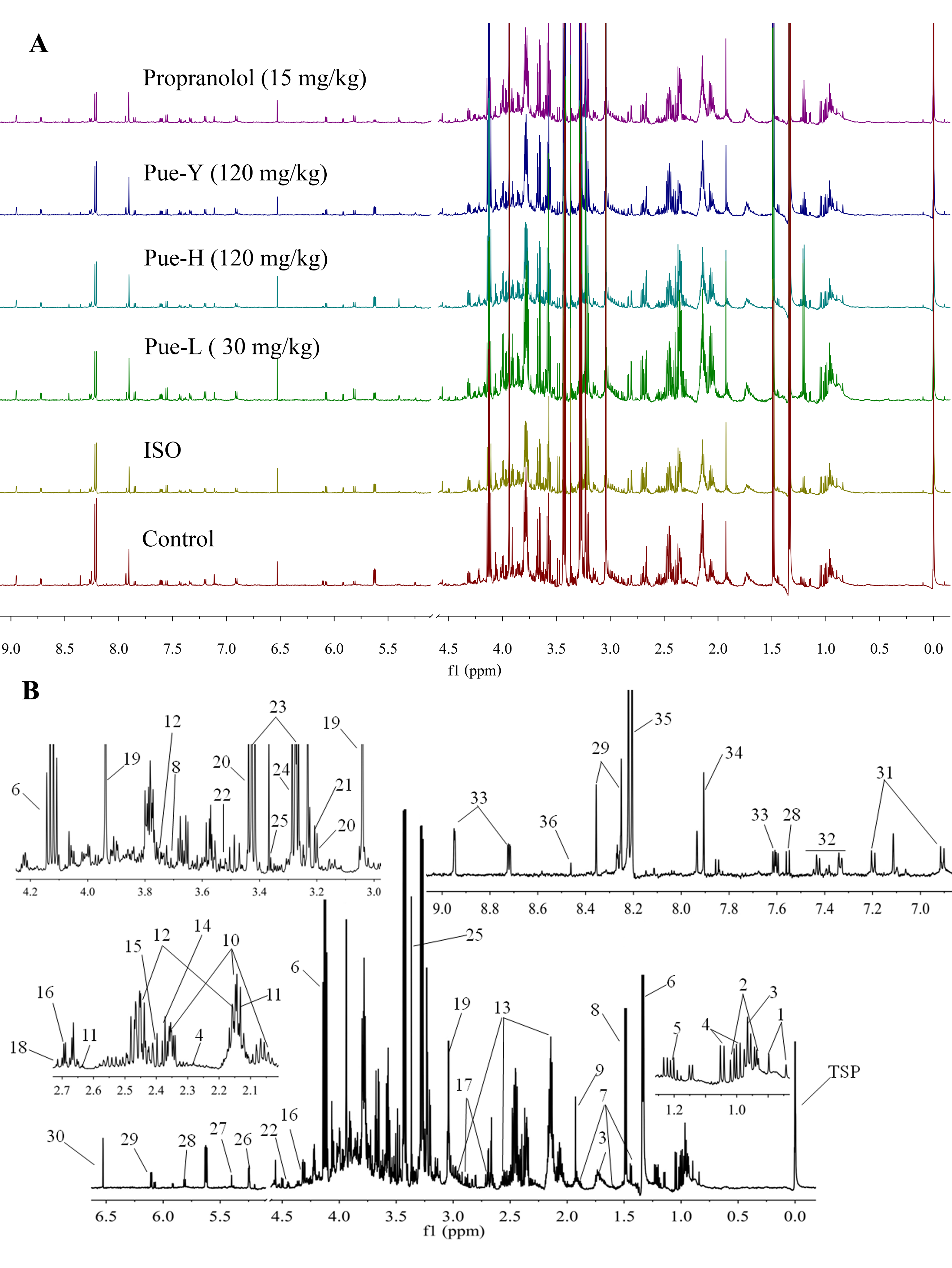
**

### Fig.S2. (A) Typical 600 MHz ^1^H NMR spectra of cardiac tissues from different groups. (B) ^1^H-NMR spectra of control rat for metabolites identified from the cardiac tissues. The numbers of peaks for metabolites in the figure were corresponded to TableS2.

### Table S2. List of the detailed NMR assignments from cardiac samples in rats

| NO. | Metabolites | δH(multiplicity) | Samples |
| --- | --- | --- | --- |
| 1 | Pantothenate | 0.90(s),0.94(s) | H |
| 2 | Isoleucine | 0.94(t),1.01(d) | H |
| 3 | Leucine | 0.96(t),1.73(m) | H |
| 4 | Valine | 0.99(d),1.05(d),2.28(m) | H |
| 5 | 3-Hydroxybutyrate | 1.21(d) | H |
| 6 | Lactate | 1.33(d),4.12(q) | H |
| 7 | Lysine | 1.45(m),1.71(m),1.90(m) | H |
| 8 | Alanine | 1.49(d),3.77(q) | H |
| 9 | Acetate | 1.93(s) | H |
| 10 | Glutamate | 2.06(m),2.14(m),2.35(m) | H |
| 11 | Methionine | 2.14(s),2.64(t) | H |
| 12 | Glutamine | 2.15(m),2.44(m),3.77(m) | H |
| 13 | GSSG | 2.15(m),2.54(m),2.95(m) | H |
| 14 | Pyruvate | 2.37(s) | H |
| 15 | Succinate | 2.41(s) | H |
| 16 | Malic acid | 2.69(dd),4.31(dd) | H |
| 17 | Aspartic acid | 2.69(dd),2.82(dd) | H |
| 18 | Dimethylglycine | 2.72(s) | H |
| 19 | Creatine | 3.04(s),3.94(s) | H |
| 20 | Choline | 3.20(s),3.43(t) | H |
| 21 | PC | 3.21(s) | H |
| 22 | GPC | 3.23(s),3.63(m),4.31(m) | H |
| 23 | Taurine | 3.27(t),3.43(t), | H |
| 24 | Betaine | 3.27(s) | H |
| 25 | Scyllo-inositol | 3.37(s) | H |
| 26 | a-Glucose | 5.24(d) | H |
| 27 | Glycogen | 5.41(m) | H |
| 28 | Uracil | 5.81(d),7.55(d) | H |
| 29 | Adenosine | 6.10(d),8.25(s),8.36(s) | H |
| 30 | Fumarate | 6.53(s) | H |
| 31 | Tyrosine | 6.91(d),7.20(d) | H |
| 32 | L-Phenylalanine | 7.34(m),7.38(m),7.43(m) | H |
| 33 | Niacinamide | 7.61(dd),8.26(d),8.72(d),8.95(d) | H |
| 34 | Xanthine | 7.90(s) | H |
| 35 | Hypoxanthine | 8.21(s),8.22(s) | H |
| 36 | Formate | 8.46(s) | H |

### Multiplicity^a^: singlet(s), doublet(d), triplet(t), doublet of doublets(dd), multiple (m), quartets(q). PC: phosphocholine; GPC: glycerol-phocholine; GSSG: glutathione disulfide; H: heart.
